# Editorial: Interplay of Infection and Microbiome

**DOI:** 10.3389/fcimb.2020.00304

**Published:** 2020-06-17

**Authors:** Wilhelmina M. Huston, Gilda Tachedjian

**Affiliations:** ^1^Faculty of Science, School of Life Sciences, University of Technology Sydney, Ultimo, NSW, Australia; ^2^Burnet Institute, Melbourne, VIC, Australia

**Keywords:** microbiota, infectious disease, next generation sequencing, pathogenesis, inflammatory

The composition and function of the animal body is now widely acknowledged to be critically linked with the numerous microscopic organisms that have a habitat within the animal host. There are numerous lines of evidence that these tiny ecosystems can be critical for the local function of each tissue site, or can act as a pathogen defense system, or in some cases can be associated with disease. There is gathering evidence that the hosts diet, genotype, and lifestyle can impact on these microbial ecosystems throughout the body. The composition of the microbiota, or microbiome of the host, from humans to a myriad of animal hosts, has been the focus of recent research.

More and more it is becoming apparent that interactions between pathogenic infections and the local microbial community at the infection site is an important factor in the outcomes of diseases across humans and various animals.

The topics in this Research Topic issue span across the human and animal sphere and explore the infection and microbiota interplay through three major themes. The first theme focusses on the interactions between microbiome, metabolites and host responses; the second theme addresses microbiome compositions as predictive indicators or associated factors in cancers; and the final theme addresses the functional molecular and genomic knowledge that is now emerging on key microbiome players.

## Is it the Microbiome Composition or the Resulting Metabolome That is Most Influential?

A priority to microbiome researchers and those considering probiotic prophylaxis is the concept of a pre-disposing immune environment due to the status of the local microbiome and how that may then interplay into the infection.

The role of L-tryptophan as a keystone metabolite that is critical for host nutritional and immune regulation, but also as a target for the gut microbiome manipulation to orchestrate the host immune status is comprehensively reviewed (Gao et al.). The review explores the molecular processes and metabolic compounds involved, identifies the knowns and unknowns for the major metabolites in the host and microbial pathways, outlines lifestyle and environmental factors that may influence these pathways, and outlines the diseases where this host and microbiome interplay around a keystone metabolite may be important.

In the article by Delgado-Diaz et al. the influence of metabolic constituents associated with optimal and non-optimal vaginal microbiome compositions on cervicovaginal epithelial cellular responses was explored. The authors examined the inflammatory response profile of epithelial cells exposed to the metabolic profile associated with a bacterial vaginosis microbiome (non-optimal) compared to that of a lactobacilli dominated (optimal) vaginal microbiome. They found that short chain fatty acids, at levels observed in women with bacterial vaginosis, were associated with dysregulation of the immune response to inflammatory stimuli that may explain, in part, the pro-inflammatory cytokine and chemokine profile observed in women experiencing bacterial vaginosis.

Further to this insight into the disease burden of bacterial vaginosis, an approach to define a standardized (“universal”) donor screening for a vaginal microbiome transplant has been outlined in the issue by DeLong et al.. Microbiome transplants, whilst limited success has been reported to date, are viewed as a likely more successful methodology to create a longer term change in a microbial ecosystem than administration of a probiotic, and particular in the case of this polymicrobial condition.

Finally, in this theme, the current knowledge of how the host gut microbiota interplays into the immune responses to nematodes and protozoa in mice and mosquito vectors is reviewed (Yordanova et al.), presenting a fascinating summary of the structural and physiological environments the parasites have to adapt in distinct life-cycle phases.

## Could the Microbiome be an Indicator or Predictive Biomarker for Cancer?

The role of microbiome signatures as a potential indicator of oral and oropharyngeal cancers was explored in a case-control study of 83 participants conducted by Lim et al.. In this study, which requires further validation, the findings indicated that profiling the oral rinse microbiome had 100% sensitivity and 90% specificity to predict these cancers.

Differences in the bacterial diversity profiles and also higher representations of certain bacterial genera were identified when microbial profiles of mid-stream urine samples of males with bladder cancer was compared to controls. This study (Wu et al.) of 60 men in total has provided preliminary evidence that there may be a microbiome profile consistent with the presence of bladder cancer.

A study presented in the issue comparing the profile of the mucosal microbiome from the gastrointestinal surfaces of patients with gastric antrum or duodenal ulcers identified that there was a higher proportion of Helicobacter in those with the gastric ulcers (Chen et al.). There were also a number of species that were over-represented in the specimens from the duodenal ulcers than the gastric sites, information which may have relevance in future management of this condition as the composition of the local microbiome could interplay into the pathology (in addition to the causation by *Helicobacter*).

## Insights Into Key Microbiome Players

Intestinal helminth infections have been associated with alterations in the gut microbiome; however, whether this directly relates to helminths or is an indirect consequence of the immunological and metabolic response to the infection has not been certain. Given helminths have to compete with the gut microbiome to establish the infection, Midha et al.. have examined the antibacterial properties of the intestinal roundworm *Ascaris suum*, and identified excretory and secretory factors with broad spectrum antimicrobial factors. These findings demonstrate a specific functional impact on the microbiome as part of the pathogenic process for at least this helminth.

*Campylobacter concisus*, an organism known to colonize the oral cavity of humans, but has been identified as over-represented or dominant in a number of conditions (gastroenteritis, Barrett's Esophagus, and gingivitis or periodontal diseases), although as this comprehensive review points out many of the studies were underpowered or did not include appropriate controls meaning association with these conditions cannot yet be concluded. However, of particular interest is irritable bowel disease, where over-representation of *C. concisus* in the oral cavity has been reported in more than one study. The review also presents genomic analysis of the species, pathogenic mechanism, pathology and inflammatory associations, and anti-microbial resistance properties. The review (Liu et al.) also shares insights into other potentially important members of the genus.

The capacity of Lactobacilli strains (and their secretions) isolated from fecal samples from healthy children to prevent or disperse biofilms formed by *Vibrio cholera* and *Vibrio paraheamolyticus* was analyzed Kaur et al. to investigate their preventative or therapeutic potential against this diarrheal disease. The authors found that the low pH elicited by the strains was the main mechanism of antimicrobial activity against the Vibrio. However, in a strain specific manner, some culture supernatants where able to inhibit biofilm formation or disperse biofilms, even when the pH was neutralized.

## Conclusions

The evidence is strong that the microbiome, metabolic profiles, and interplay with the host are determining factors in infection risk or potentially pathology associated with infection. However, the precision medicine applications that could emerge, and the therapeutic interventions remain in the distant future until greater understanding of the physiological and molecular features at play can be more rigorously and thoroughly profiled. This issue presents some up to date and promising data and reviews on this topic that point to the microbiome being a critical focus for on-going research into infection.

## Author Contributions

All authors listed have made a substantial, direct and intellectual contribution to the work, and approved it for publication.

## Conflict of Interest

The authors declare that the research was conducted in the absence of any commercial or financial relationships that could be construed as a potential conflict of interest.

